# Reply to Dr. Taft

**Published:** 1893-11

**Authors:** 


### Domestic Correspondence.

### REPLY TO DR. TAFT.

BOSTON, September 12, 1893.

TO THE EDITOR:

SIR,—I read a paper before the Massachusetts Dental Society, at the Annual Meeting, June 8, 1893, on "Amalgam as a Filling-Material." It was published in the INTERNATIONAL DENTAL JOURNAL of August. Dr. C. H. Taft, of Chicago, published a letter, in the September number, apparently intended to be a criticism of my paper, which demands a reply. If I failed to do this, the profession outside of the Massachusetts Dental Society would naturally be led to believe that Dr. Taft had been abused. I will not take up a great deal of your valuable space in noticing all he has written, as that which is of any account can be summed up in a few words, and when the incorrect statements are taken out, there is very little worthy of reply. Dr. Taft says in his letter, "Now, doctor, allow me to suggest that, considering the facts were all ready and waiting for a hearing, and which, partly by your own vote and influence, as is well known, were refused a hearing, such sentiments as the ones I have quoted from your paper are neither manly nor sincere." This statement is untrue, as I did not vote against having his paper read. He also says in his letter, "Before a member in good standing is again invited to read a paper before the Massachusetts Dental Society and refused a hearing because he insists upon the natural right and privilege of every man to say for himself, without assistance from others, what shall be the title of any subject upon which it is always his prerogative to write, it is to be hoped the Society will have learned a lesson in the rules which govern fair play." Dr. Taft was not invited to read this paper referred to on Amalgam by the Massachusetts Dental Society, neither did the Executive Committee invite him. This Committee met several times during the winter of 1892 and 1893 to prepare for the Annual Meeting of the Society. After completing its work, at a meeting May 15, 1893, only a little more than three weeks before the Annual Meeting, June 8, a friend of Dr. Taft asked the Executive Committee for the privilege of having a paper from Dr. Taft read before the Massachusetts Dental Society at the Annual Meeting in June. Whether this was at the request of Dr. Taft or not I am unable to say.

I will submit the following statement from the Secretary of the Executive Committee of the Massachusetts Dental Society.

To Edward Page, M.D., D.M.D.:

Dear Doctor,—In the matter of a paper written by Dr. Charles H. Taft, of Chicago, upon the subject of "Injurious Effects of Amalgam Fillings," and presented to the Executive Committee of the Massachusetts Dental Society by Dr. E. O. Kinsman for consideration, May 15, 1893, the following vote was passed:

"That, owing to the programme being completed, it is the sense of the Committee that it is inexpedient to accept the paper."

Unanimously voted.

Waldo E. Boardman, D.M.D.,
*Secretary of the Executive Committee*
*Massachusetts Dental Society.*

Boston, September 11, 1893.

The paper of Dr. Taft, it will be observed, was not rejected on account of the title, as he has stated.

Edward Page, M.D., D.M.D.

[Remarks.—Both sides of this question having now been heard, we cannot extend this controversy further. It seems to have resolved itself into a question of fact, which should be settled privately by the parties most interested.—Ed.]

